# Safety and Efficacy of External Nasal Dilator Strips with N95 Respirator Masks by Emergency Department Personnel

**DOI:** 10.51894/001c.30215

**Published:** 2022-02-24

**Authors:** Ariel Hawley, Mitchell Rozman, Matthew Hysell

**Affiliations:** 1 Emergency Medicine Spectrum Health - Lakeland; 2 Internal Medicine McLaren Greater Lansing

**Keywords:** covid-19, external nasal dilator strips, n95 respirator, emergency department

## Abstract

**INTRODUCTION:**

The coronavirus disease 2019 (COVID-19) pandemic has prompted increased use of personal protective equipment (PPE) to maintain the health and safety of caregivers. This study was conducted in 2020 to evaluate the safety and efficacy of external nasal dilator strips (ENDS) coupled with N95 respirators in a sample of community hospital emergency department personnel.

**METHODS:**

After obtaining written consent, the authors tested participants’ response to exercise (i.e., walking up 10 flights of stairs) while wearing an N95 respirator, both with and without an ENDS. The authors measured participants’ heart rate and respiratory effort responses over four minutes following their exercise trial. A convenience sample of these personnel also repeated their respirator fit testing while wearing an ENDS with the N95 style they had previously been fitted for.

**RESULTS:**

A total of N = 50 participants were enrolled. Peak heart rate while wearing an ENDS was 125 beats per minute (BPM) with the ENDS versus 130 BPM without (p = 0.21). The Borg Exertion Score while wearing an ENDS peaked at 13 with the ENDS versus 14 without (p = 0.08). However, when subjects were surveyed before and after the trial upon whether they would consider using an ENDS beneath their N95 using a scale of 1-5, their interest in this significantly increased (p = 0.004). Four of the 13 (31%) participants who completed repeated fit testing while wearing the ENDS beneath their N95 respirator failed the repeat testing.

**CONCLUSIONS:**

These results first suggest that a sizable proportion of ED personnel may fail N95 fit testing while wearing an ENDS beneath the N95 mask for which they had been previously fitted. Although providers’ subjective interest in use of ENDS increased, these results also demonstrate that use of an ENDS beneath an N95 respirator may not significantly increase exercise tolerance.

## INTRODUCTION

The coronavirus disease 2019 (COVID-19) pandemic has prompted the increased use of personal protective equipment (PPE) to maintain the health and safety of healthcare workers. N95 respirators (which block at least 95 percent of very small (0.3 micron) test particles) provide barriers aimed at preventing the transmission of aerosol transmissible diseases to healthcare personnel. N95 respirators must be tight fitting to maintain a proper seal, which often leads to some undesirable side effects (e.g., constricting the nasal passages, in some cases skin breakdown).^1,2^

Given the widespread use of PPE during the COVID-19 pandemic, Yildiz et. al., examined the use of external nasal dilator strips (ENDS) as prophylactic dressings under PPE to prevent skin injuries in healthcare workers.^3^ This 2021 study group found a significant reduction in skin injuries with use of ENDS beneath masks and fewer reported cases of breathing discomfort.^3^ However, it should be noted that the safety of using ENDS under N95 respirators was not evaluated in this study.

Although the efficacy of ENDS when used with N95 respirators has not been previously demonstrated, their use within sleep medicine and sports medicine has been well-studied and demonstrated to facilitate breathing in sleep, sports, and sinonasal disease.^4^ Current literature demonstrates that ENDS are an effective means to prevent and treat sleep disorders, snoring, and nasal congestion both through subjective and objective indicators.

Although it has been demonstrated that ENDS do not reduce work of breathing while exercising at 70% maximum rate oxygen used during exercise (VO_2_ max), other objective markers have shown improvement with application of ENDS.^5^ For example, Deyak et. al., demonstrated in 1998 that Division I collegiate hockey players had significantly faster skate times, shorter recovery periods to baseline heart rate (HR), and lower blood lactate levels when using ENDS.^6^

ED personnel frequently have exertional components to their work (e.g., administration of cardiopulmonary resuscitation (CPR) or orthopedic reductions). N95 respirators have been recommended by the Center for Disease Control (CDC) while performing clinical duties during which providers come in close contact with patients suspected or known to be infected with COVID-19.^1^ To our knowledge, there was no prior published research at this time evaluating whether: a) ENDS are safe and/or effective when worn in conjunction with N95 respirators, or b) how ENDS use might moderate dyspnea and/or HR during exercise while wearing an N95.

### Purpose of Study

The primary objective of this study was to examine if use of an ENDS with the N95 respirator increased exercise tolerance, measured by a validated exertion score, peak HR, and time to return to baseline HR. The authors’ secondary objective was to evaluate whether N95 respirators retained their function when used in conjunction with ENDS.

## METHODS

### Study design and data collection

After receiving “expedited” approval from the Spectrum Health Lakeland IRB, this study was conducted in 2020 at a community-based ED with an emergency medicine residency program. Prior to their written consent to participate in the study, all participants had been separately fitted for and issued N95 respirators. The approved N95 respirators used in this study were either Surgical Mask 1860 or 1860S or 3M Particulate Respirator 9210+, N95.^7,8^

During exercise testing, participants used score sheets to first record their baseline HR and complete the Borg’s Rate of Perceived Exertion (RPE) scale rating at rest.^9^ The RPE is a validated scale used in exercise research to assess degrees of dyspnea during exercise.^9^ Scores range from 6 (“No Exertion”), 7-9 (“Light Breathing”), 10-12 (“Deeper breathing but subject is still conversational”), 13-14 (“Conversation is difficult”), 15-16 (“Subject is uncomfortable”), 17-19 (“Breathing deep and forceful”), up to 20 at maximal exertion. Heart rates were measured and recorded by the participants palpating their radial pulse and counting HR over a 15-second period.

As a surrogate measure of exertion during ED clinical duties, the authors asked participants to complete two exercise trials to investigate whether ENDS use improved exercise tolerance while wearing an N95 respirator. In one trial, participants performed the stair climb exercise with an N95 respirator alone. In the other trial, they performed the same stair climb exercise while wearing an ENDS underneath the N95 respirator. Participants were alternately randomized with regards to whether they completed the trial with or without the ENDS first. Hand hygiene was utilized per standard protocol when replacing masks.

The ENDS used during the study were generic Walgreens brand tan extra-strength nasal strips. During each trial, groups of four-to-six participants joined an investigator to climb 10 flights of stairs (i.e., 100 steps). Upon completion of 100 steps, participants immediately recorded their HR and RPE scale rating (Time Zero). Heart rates and RPE scale ratings were subsequently recorded every 60 seconds for four minutes (i.e., total of five time points).

After the completion of both trials, participants were asked how likely they were to use an ENDS with the N95 respirator in the future. Data were recorded on de-identified scoresheets and were anonymously collected.

A convenience sample subset of participants also completed qualitative respiratory fit testing while wearing an ENDS beneath their pre-study assigned and fitted N95 respirator. Qualitative respirator fit testing is a pass/fail test method that uses sense of taste or smell to detect leakage into the respirator facepiece.^10^

### Study Sample

All study participants were ED personnel who had previously been fitted for and issued N95 respirators. Inclusion criteria were personnel working in the ED, (i.e., attendings and resident physicians, mid-level providers, nurses, techs, and fourth year medical students). Exclusion criteria consisted of self-reported medication use that could affect HR, or physical inability to perform the stair climbing exercise.

### Data analysis and outcomes

The primary outcomes of interest were peak HR, time to return to baseline HR, and perceived RPE scale ratings in response to exercise while wearing a fitted N95 respirator with and without ENDS use. We secondarily examined successful respirator fit testing while wearing the ENDS underneath the N95 respirator.

The third author (MH) performed statistical analyses using a series of two-tailed Student’s t tests to evaluate for differences between HRs and Borg’s RPE scores at each time point, in addition to the visual analog scale regarding participant’s interest in using ENDS in the future. These analyses were performed using free calculators available from www.socscistatistics.com.

## RESULTS

### Sample Characteristics

A total of N = 50 participants were enrolled in this study. Of these, 26 (52%) were female. The median age was 31 years, (interquartile range (IQR) 21 - 48 years). Of the 50 study participants, 25 (50%) were resident physicians, 14 (28%) were registered nurses, five (10%) were fourth year medical students, 5 (10%) were ED techs, and one (2%) was an attending physician.

### Exercise Tolerance results

Following randomization, 27 (54%) participants completed the initial exercise without using an ENDS whereas 23 (46%) completed their initial exercise with an ENDS. In the subgroup of those who initially climbed without an ENDS, their peak HR was 129 beats per minute (BPM). Their peak HR reduced to 127 BPM while using the ENDS during the second climb.

**Figure 1.**
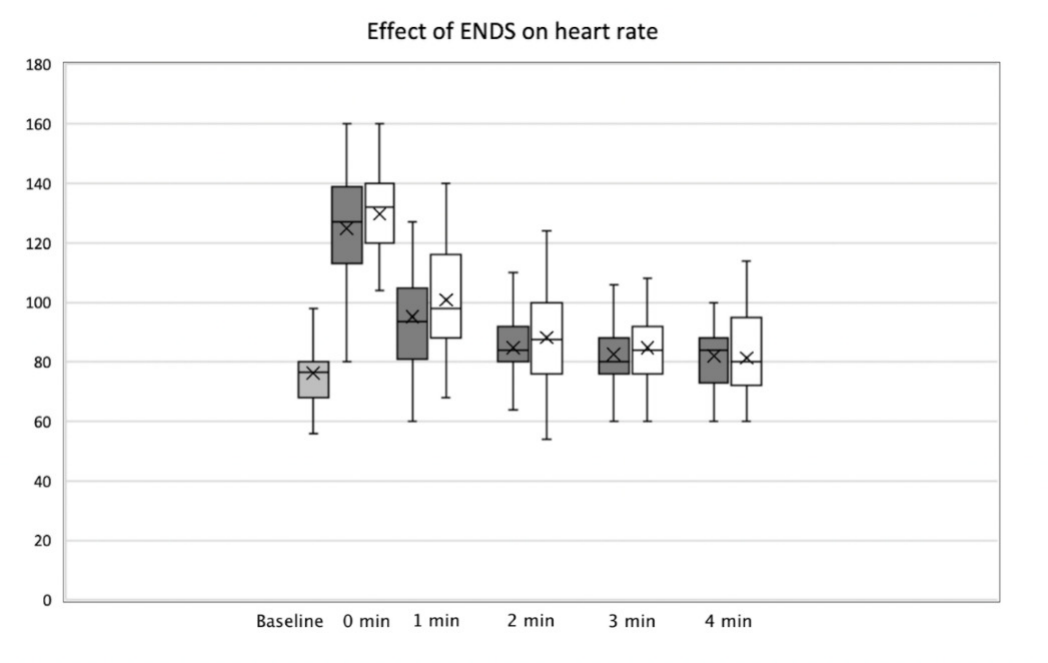
Effect of ENDS on heart rate at Baseline, Time Zero, One minute, Two minutes, Three minutes, and Four minutes after completion of stair climb. Shaded bars = patients with ENDS, open bars = patients without ENDS.

In the subgroup who first climbed with the ENDS, the peak HR was 122 BPM compared with 131 BPM in their subsequent climb without. The pooled average peak HR of climbers using the ENDS was 125 BPM, and the peak HR average of climbers without using the ENDS was 130 BPM. Although this demonstrated a difference in average peak HR of 5 BPM, this was not statistically significant (p = 0.21). By Minute 3, both arms of the study had essentially no difference in HR (Figure 1).

**Figure 2.**
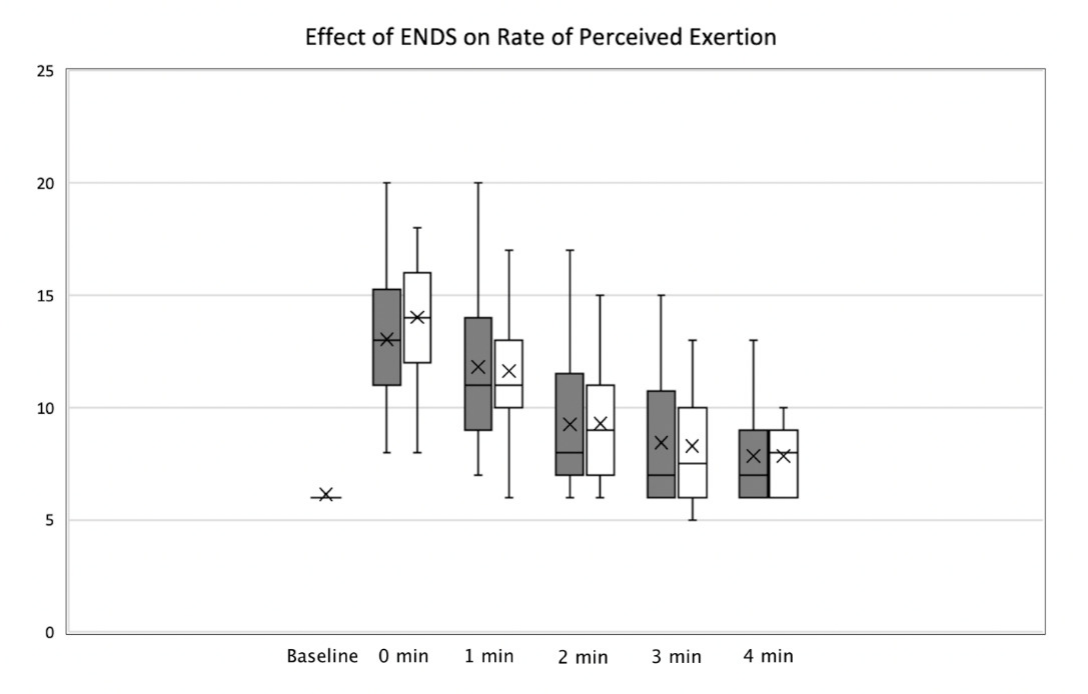
Effect of ENDS on RPE at Baseline, Time zero, One minute, Two minutes, Three minutes, and Four minutes after completion of stair climb (p = 0.08). Shaded bars = patients with ENDS, open bars = patients without ENDS.

Similarly, there was a slight difference in RPE exertion scores. Exercise trials using the ENDS averaged a peak score of 13, while the average peak score was 14 in trials without the ENDs. This difference approached, but did not reach, statistical significance (p = 0.08). There was essentially no difference in average exertion scores 1+ minute after exercise (Figure 2).

### Subjective assessment of ENDS use with N95

Before their initial stair climb, participants were first asked how likely they would be to wear an ENDS. They were also asked the same question following the trials, rating their response on a Likert 1 to 5 scale from “No Chance” (1), to “Unsure” (3), to “Certain” (5). Initial responses averaged 2.97 (SD = 0.93) and final responses averaged 3.58 (SD = 0.79), which was significant (p < 0.01).

### Fit testing results with ENDS

The secondary outcome, successful fit testing using an ENDS in conjunction with their earlier-fitted N95 respirator per hospital policy, was achieved in only nine out of 13 (69%) of participants. A convenience subgroup of consented participants underwent repeat fit testing while wearing an ENDS underneath their assigned N95 respirator on a separate occasion from their participation in the exercise trials. Four (31%) participants failed fit testing while using an ENDS. One of these four participants had been fitted for a 3M Health Care Particulate Respirator Surgical Mask 1860 and one had been fitted for the 3M Particulate Respirator 9210+, N95 (the other two participants did not specify which respirator was worn).

## DISCUSSION

Investigators have long evaluated the N95 respirator’s effects upon discomfort from altered cerebral hemodynamics and breathing resistance.^11^ It was our hope that use of ENDS might ameliorate some of these effects. Of the 50 participants enrolled in this study, 13 (26%) were respiratory fit tested while wearing an ENDS beneath their assigned N95 respirator. Of those 13, an alarming four participants (31%) failed the respirator fit test.

This finding of inadequate N95 fit in ENDS sample users may be due to the ENDS having caused increased nasal dilation and allowed increased passage of particulate test matter into the nostrils, not detected by the user.^12^ It is also possible that participants failed fit testing at an increased rate while wearing ENDS beneath their assigned N95 respirator due to placement of the ENDS high on the nasal dorsum, so as to interfere with the respirator seal.^12^ Due to these potential fit limitations, our higher fit testing fail rate suggests that healthcare workers who choose to wear ENDS beneath the N95 respirator should likely undergo fit testing to ensure that ENDS do not interfere with the respirator seal.

To our knowledge this is the first study evaluating the safety and efficacy of ENDS in a sample of healthcare workers. In the sports medicine world, results have been mixed. In 1999, Seto-Poon, et. al., demonstrated that athletes were able to continue nasal breathing longer while wearing ENDS although Deyak, et. al., found improved performance in college hockey players using ENDS.^6,13^ In 2001, O’Kroy, et. al., did not find increased performances as athletes pushed into higher exertion rates.^5^ It is unlikely that this difference of five BPM with ENDS use would be clinically significant, similar to the 2020 results of Overend, et. al.^14^

It is notable that some participants in our study expressed increased interest in using ENDS even though our results failed to show overall sample significant differences in HR or RPE scores. Yildiz et. al., similarly demonstrated that use of ENDS improved perceived breathing discomfort in a small sample.^3^ As dyspnea is a subjective finding, this may represent more of a psychological or placebo component from ENDS use. Alternatively, the main benefit of the ENDS may lie with improving baseline breathing and improving participants’ ability to nasally breathe while *not* working hard. ENDS have been previously shown to decrease nasal resistance and oral fraction of ventilation during sleep while also increasing sleep architecture.^15^ In 2007, Høyvoll, et al demonstrated that minimal cross-sectional areas of the anterior nasal passages and nasal cavity volumes as measured by acoustic rhinometry were increased after application of an END with efficacy like that of decongestive nose drops.^12^

### Study Limitations

First, most of our participants in this smaller convenience sample were young individuals who appeared to be in relatively good health. Second, human error must be considered, as participants self-applied their ENDS under their N95 respirator and measured their own HR. It is possible that some participants may have placed their ENDS higher on their nasal dorsum (i.e., bridge of their nose) where it would be less likely to open the nasal passages but more likely to interfere with the seal of the respirator. Finally, we recognize that our surrogate stair climbing measure of exertion did not exactly mirror the typical workday of ED providers.

## CONCLUSION

Our results indicate that ENDS use may sometimes compromise the fit integrity of N95 respirators. Healthcare personnel should probably successfully pass a second respirator fit test before using an ENDS. Future studies of ENDS with N95 respirators in larger samples are needed to examine the simultaneous use effects of these devices on both baseline breathing and exercise tolerance.

### Conflicts of interest

The authors report no conflicts of interest.
